# Detection of *Hanseniaspora opuntiae* in anovaginal samples of pregnant women in Rio de Janeiro, Brazil—a case report

**DOI:** 10.3389/fcimb.2024.1394663

**Published:** 2024-05-30

**Authors:** Tatiane Nobre Pinto, Laura M. A. Oliveira, Gisela L. da Costa, Natália Silva Costa, Elaine Cristina Francisco, Tatiana C. A. Pinto, Manoel M. E. Oliveira

**Affiliations:** ^1^ Laboratório de Cocos Patogênicos e Microbiota, Instituto de Microbiologia Paulo de Góes, Universidade Federal do Rio de Janeiro, Rio de Janeiro, Brazil; ^2^ Laboratório de Taxonomia, Bioquímica e Bioprospecção de Fungos, Instituto Oswaldo Cruz, Fundação Oswaldo Cruz, Rio de Janeiro, Brazil; ^3^ Laboratório Especial de Micologia, Universidade Federal de São Paulo, São Paulo, Brazil

**Keywords:** *Hanseniaspora*, *Streptococcus agalactiae*, opportunistic pathogens, apiculate yeasts, MALDI-TOF MS

## Abstract

In this study, we report the first isolation of *Hanseniaspora opuntiae* obtained from four pregnant women in Brazil. Clinical isolates were obtained from four samples taken between 35 and 37 gestational weeks, as part of the routine antenatal care for maternal colonization screening for *Streptococcus agalactiae* group B. The patients were immunocompetent, with two of them diagnosed with gestational diabetes mellitus. Species identification was performed by MALDI-TOF MS and rDNA sequencing. While *Hanseniaspora* species have not traditionally been considered a typical opportunist pathogen, our findings emphasize the importance of investigating and screening for *Hanseniaspora* in pregnant populations, highlighting *H. opuntiae* as a potential agent of human infections.

## Introduction

The genus *Hanseniaspora* is part of the *Saccharomycetaceae* family, which is characterized by apiculate yeasts with bipolar budding cells and globose, warty ascospores (Cadez and Smith, 2011). Currently, the genus comprises approximately 20 species that can be divided into two major clades: the “fermentation clade,” known for its robust fermentation capability, and the “fruit clade,” consisting of species more adapted to fruit colonization (Čadež et al., 2019; van Wyk et al., 2024). While members of *Hanseniaspora* spp. have been extensively studied for their ability to enhance the sensory profile of wines and beer (Martin et al., 2018; Burini et al., 2021; Zhang et al., 2023), there is a significant gap in their clinical relevance.

Although rare, *H. uvarum* and *H. opuntiae* have been documented to play a pathogenic role in both immunocompetent and immunocompromised patients, with reports of superficial mycoses, gastroenteritis episodes, oral injuries, and invasive infections (Emmanouil-Nikoloussi et al., 1994; García-Martos et al., 1998; Karimi et al., 2015; González-Abad and Sanz, 2018; Jankowski et al., 2018). Recent studies have highlighted an increase in yeast colonization during pregnancy, and vaginal fungal infections in pregnant women have been associated with increased pregnancy complications (Disha and Haque, 2022). Notably, a significant rise in *H. pseudoguilliermondii* and *H. meyeri* has been noted in pregnant women with gestational diabetes mellitus (GDM) (Wu et al., 2022), emphasizing the necessity for a deeper understanding and assessment of *Hanseniaspora* species occurrence in pregnant individuals. In this study, we report the co-isolation of *Hanseniaspora* opuntiae and *Streptococcus agalactiae* in two pregnant women with a history of GDM who went to the Teaching Maternity of the Universidade Federal do Rio de Janeiro, Brazil.

## Description of four cases

The four clinical isolates of *Hanseniaspora* were recovered from four pregnant women undergoing routine antenatal care procedures and maternal rectovaginal colonization surveillance for *S. agalactiae* (Group B or GBS) at the Teaching Maternity of the Federal University in 2020. A questionnaire was used during medical examinations to collect clinical data from the patients, who were aged between 13 and 37 at the time of collection. One patient reported a history of urinary tract infection during pregnancy plus vaginal discharge, and the use of the antibiotic cephalexin as treatment (**Table 1**). While two other patients reported the use of the antibiotic cephalexin during pregnancy, the specific period of usage was not documented. All patients were immunocompetent, with two of them having GDM.

**Table 1:** Demographic data and laboratory findings of the four *Hanseniaspora opuntiae* clinical isolates obtained from pregnant women.

Demographic and laboratory data	Patient code			
	13918	13922	13951	13964
Age (years)	36	20	13	37
City of birth	RJ	RJ	RJ	RJ
Vaginal discharge during pregnancy	No	Yes	Uninformed	No
Previous premature childbirth	No	No	No	No
Urinary tract infection	No	Yes	No	No
Use of antibiotics	No	Cefalexin	Cefalexin	Cefalexin
Underlying disease	No	Gestational diabetes mellitus	No	Gestational diabetes mellitus
Neonatal death history	No	No	No	No
GBS detection	+	−	−	+
MALDI TOF identification/score value	*H. opuntiae*/1.763	*H. opuntiae*/1.763	*H. opuntiae*/1.763	*H. opuntiae*/1.763
Sequencing identification	*H. opuntiae*	*H. opuntiae*	*H. opuntiae*	*H. opuntiae*

Urinary tract infection and antibiotic use were factors considered at the time of sampling. RJ, Rio de Janeiro; GBS, *Streptococcus agalactiae* group B; +, positive; − negative.

The four samples were collected from the anus and vagina (anovaginal), during the 35–37 weeks of gestation, using a single swab for both the anal and vaginal regions while avoiding the cervix, in accordance with current guidelines (Filkins et al., 2020; Costa et al., 2022). Subsequently, the swabs were preserved in skim milk, tryptone, glucose, and glycerin (STGG) transport medium at −20°C. An aliquot of the STGG medium containing the swabs was inoculated onto blood agar and Sabouraud dextrose agar (BD Difco, Franklin Lakes, USA) plates supplemented with 0.05 g/L chloramphenicol. The plates were then incubated at 35°C for 48 h. Following the incubation period, colonies of *S. agalactiae* were observed in two clinical samples and yeast growth was observed from all four clinical samples. Yeast identification was conducted by MALDI-TOF MS using the Bruker Daltonics platform (Bruker Daltonics, Bremen, Germany). Protein extraction was performed according to the manufacturer’s recommendations with few modifications (Wang et al., 2021; Pinto et al., 2022). The isolates were tested in triplicate and data analysis was performed using the Biotyper™ 3.1 software (Bruker Daltonics), with log score ≥ 2.0 considered high confidence. Using the Bruker database, the four isolates were identified as *H. opuntiae* with a score ranging from 1.761 to 1.878 (**Table 1**).

The species was identified by sequencing the ITS1–5.8S-ITS2 rDNA region using a new DNA extraction method developed by our team. This methodology is an adaptation of the colony PCR technique described by Mirhendi et al. (2007) and is described for the first time in this paper. In our methodology, after 48 h of growth at 30°C, a small amount of each isolate was selected (approximately 10^6^ yeast cells), transferred to PCR tubes, and manually smeared using a 10-μL pipette tip. The tubes were then heated in a microwave for 90 s at maximum power and immediately cooled to 0°C. The PCR mix was prepared to a final volume of 50 μL, containing 1 μg of DNA and 25 ng/μL of the ITS1 (5′-TCCGTAGGTGAACCTGCGG-3′) and ITS4 (5′-TCCTCCGCTTATTGATATGC-3′) primer pair (Van Gerrits den Ende and de Hoog, 1999; Pinto et al., 2022; Costa, 2023; Oliveira et al., 2023). Sequencing was carried out using the ABI-3730 (Applied Biosystems) at the Network Technological Platforms of Fiocruz. Sequencing editing was performed using CodonCode Aligner (Genes Code Corporation, Ann Arbor, United States). The consensus sequences were cross-referenced with those available in the NCBI database (http://ncbi.nlm.nih.gov) using the BLASTn tool. Only sequences with a percentage of identity and sequence coverage of ≥98% and an E-value of <10^−5^ were considered. Phylogenetic analysis was performed by using the Neighbor-Joining method based on the Kimura two-parameter model with 1,000 bootstrap pseudo replicates in MEGA X (Kimura, 1980; Felsenstein, 1985; Saitou and Nei, 1987; Cadez et al., 2003; Kumar et al., 2018).

**Figure 1:**
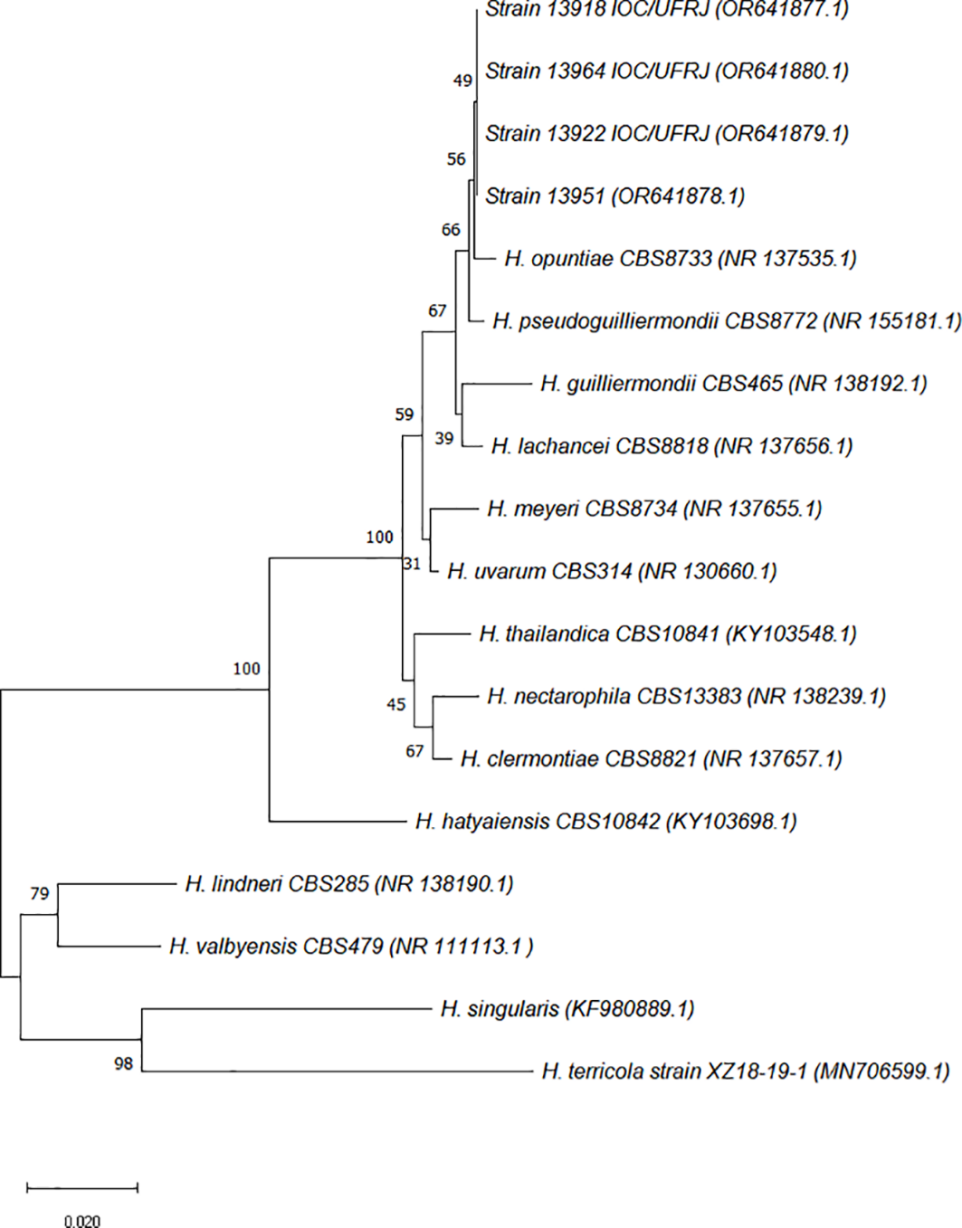
The phylogenetic analysis was inferred using the Neighbor-Joining method (Saitou and Nei, 1987). The optimal tree is shown. The percentage of replicate trees in which the associated taxa clustered together in the bootstrap test (1,000 replicates) is shown next to the branches (Felsenstein, 1985). The tree is drawn to scale, with branch lengths in the same units as those of the evolutionary distances used to infer the phylogenetic tree. The evolutionary distances were computed using the Kimura 2-parameter method (Kimura, 1980). These distances are measured in the number of substitutions for each site. Four clinical isolates from our investigation and 14 sequences of reference strains retrieved from GenBank data formed the 18 nucleotide sequences that were analyzed. There were a total of 719 positions in the final dataset. Evolutionary analyses were conducted in MEGA X (Kumar et al., 2018).

During the BLASTn analysis, the sequences of the four clinical isolates exhibited significant similarity to *H. opuntiae* and *H. meyeri*, with 100% coverage/identity and an E-value of 0.0 for both species. Through phylogenetic analysis, four isolates were accurately identified as *H. opuntiae* (**Figure 1**). The ITS rDNA sequences were deposited in the GenBank database under accession numbers OR641877 to OR641880. This study was approved by the research ethics committee of the Clementino Fraga Filho University Hospital of the Federal University of the State of Rio de Janeiro (CAAE 43389321.9.0000.5257). All patients were granted free and informed consent.

## Discussion

In the present study, the MALDI-TOF MS proved to be a reliable tool for identifying *Hanseniaspora* spp. exhibiting high-confidence log scores for genus-level identification (1.761–1.878). Despite the lack of success in providing species characterization with a high-confidence log score (≥2.0) among our isolates, the identification of *Hanseniaspo*ra spp. at the species level by using the Bruker platform has been observed in previous studies with promising results for *H. guilliermondii*. We highlight here the relevance of optimizing commercial databases with diverse reference protein spectra of *Hanseniaspora* species (Cassagne et al., 2016).

Based on our phylogenetic analysis, the four clinical isolates were accurately identified as *H. opuntiae*. However, the limited availability of sequences from *H. opuntiae* and from its closely related species, such *H. lachancei*, *H. pseudoguilliermondii*, and *H. urvarum*, may pose a potential challenge for the correct characterization of *H. opuntiae* in routine laboratories, even when using molecular investigation methods. Conventional physiological assays are not able to distinguish *H. opuntiae* from *H. meyeri*, *H. uvarum*, or *H. guilliermondii*, but investigation of 2-keto-d-gluconate as a single carbon source may be helpful to distinguish *H. lachancei* from other *Hanseniaspora* species, including *H. opuntiae* (Cadez et al., 2003).

Although the genus *Hanseniaspora* has not been considered a typical opportunist pathogen, its species have been documented in different spectra of human mycoses (Emmanouil-Nikoloussi et al., 1994; García-Martos et al., 1998; Karimi et al., 2015; González-Abad and Sanz, 2018; Jankowski et al., 2018). To the best of our knowledge, this is the first report in Brazil of *H. opuntiae* isolated from anovaginal samples from pregnant women. According to (Wu and colleagues (2022)), the increased prevalence of the genus in the gastrointestinal microbiome of pregnant women may be related to GDM (Costa et al., 2022). Similarly, in our study, two isolates of *H. opuntiae* were recovered from pregnant women with the same condition. Elevated blood glucose levels may represent a potential risk factor for infections caused by this species, given the evolutionary adaptation of *H. opuntiae* to sugar-rich environments (phylogenetic fruit clade).

We were unable to conduct antifungal susceptibility tests on the clinical isolates and to continue laboratory monitoring of the patients to track the colonization/infection profile of *Hanseniaspora*. However, the isolation of *Hanseniaspora* species in anovaginal samples provided by this study opens discussions regarding non-*Candida* yeasts as a potential pathogen implicated in vaginal infections among this population. In conclusion, our findings emphasize the importance of investigating and screening for *Hanseniaspora* spp. in pregnant women, with *H. opuntiae* as a potential agent of human infections. Further research are needed to explore the genus comprehensively, elucidating its natural history and its health implications in pregnant women and diabetic hosts.

## Data availability statement

The datasets generated during the current study are available in the GenBank repository (https://www.ncbi.nlm.nih.gov/nuccore) under accession numbers OR641877, OR641878, OR641879.1, and OR641880.1.

## Ethics statement

The study was approved by the Research Ethics Committee of Clementino Fraga Filho University Hospital of the Federal University of the State of Rio de Janeiro (CAAE 43389321.9.0000.5257).

## Author contributions

TP: Formal analysis, Investigation, Methodology, Writing – original draft. LO: Methodology, Visualization, Writing – review & editing. Gd: Supervision, Visualization, Writing – review & editing. NC: Formal analysis, Investigation, Methodology, Writing – original draft. EF: Methodology, Visualization, Writing – review & editing. TP: Conceptualization, Writing – original draft, Writing – review & editing. MO: Conceptualization, Formal analysis, Funding acquisition, Investigation, Methodology, Project administration, Resources, Supervision, Visualization, Writing – review & editing.
